# Identification of *qGL3.5*, a Novel Locus Controlling Grain Length in Rice Through Bulked Segregant Analysis and Fine Mapping

**DOI:** 10.3389/fpls.2022.921029

**Published:** 2022-06-15

**Authors:** Lan Wang, Yang Liu, Haiyan Zhao, Yuebin Zheng, Feng Bai, Sicheng Deng, Zhixiong Chen, Jinwen Wu, Xiangdong Liu

**Affiliations:** ^1^State Key Laboratory for Conservation and Utilization of Subtropical Agro-Bioresources, South China Agricultural University, Guangzhou, China; ^2^Guangdong Laboratory for Lingnan Modern Agriculture, Guangzhou, China; ^3^Guangdong Provincial Key Laboratory of Plant Molecular Breeding, College of Agriculture, South China Agricultural University, Guangzhou, China; ^4^Songgang Middle School, Qingyuan, China; ^5^State Key Laboratory of Vegetation and Environmental Change, Institute of Botany, Chinese Academy of Sciences, Beijing, China

**Keywords:** wild rice, grain length, BSA, fine mapping, gene cloning

## Abstract

Grain length (GL) directly affects the yield and quality of rice. Very few cloned GL-related genes are applied in production because their yield-increasing effects are not obvious, and the overall regulatory networks underlying the associated processes remain poorly understood. DNA samples from two bulk DNA pools (L-pool and S-pool) and their parents (KJ01 and Huaye 3) were subjected to high-throughput sequencing. Using bulked segregant analysis (BSA), *qGL3.5* was mapped to a 0.34-Mb “hotspot” region on chromosome 3 that contains 37 genes related to various traits. Then, *qGL3.5* was mapped to the genomic interval between the flanking markers M2 and M3 using 2786 BC_4_F_2_ individuals. Because the region from B5 to B6 was not the associated region under BSA-seq analysis, *qGL3.5* was narrowed down to the interval between B6 and M3, which spanned 24.0-kb. Of all 37 genes with non-synonymous single-nucleotide polymorphisms (SNPs) between KJ01 and Huaye 3 based on BSA-seq analysis, only one complete annotated gene, *ORF18* (Gene ID: *LOC_Os03g42790.1*) was found. *ORF18* encodes an IBR-RING zinc-finger-related protein, with one really interesting new gene (RING) and two in between ring finger (IBR) domains. The knockout of *ORF18* derived from Huaye 3 using clustered, regularly interspaced, short palindromic repeat (CRISPR)/CRISPR-associated 9 (Cas9) editing technology increased the GL of the mutant by approximately 2.2 mm. The novel locus *qGL3.5* negatively regulated GL by promoting elongation of the longitudinal cell of the grain outer glume. These results provide a new genetic resource for rice grain shape breeding and a starting point for the functional characterization of the wild rice GL gene.

## Introduction

Rice is among the most important staple crop species and feeds approximately one-third of the global population. The rice grain yield is determined by three main characteristics: grain weight, number of filled grains per panicle, and number of panicles per plant (Li N. et al., 2018). In terms of grain weight, the critical factor that affects yield is grain size, which includes grain length (GL), grain width, grain thickness, and the degree of filling (Huang et al., 2013). Among these four factors, GL contributes the most to grain weight (Lin and Wu, 2003), and previous studies have shown that thin grains have better quality (Chen et al., 1997; Shi and Zhu, 1997). In addition, GL is also an important factor in rice quality.

Grain length is a complex quantitative trait that is controlled by 2–3 or more genes (Mckenzie and Rutger, 1983). To date, at least 102 QTLs controlling GL have been located (Huang et al., 2013). At least eleven genes have been cloned, and further functional studies have been conducted. The genetic mechanism underlying the effects of these genes has been elucidated and is very complex, and their modes of regulation are also diverse. *qGL3/GL3.1* encodes a PPKL family of serine/threonine phosphatases and restricts cell division in spikelet hulls by dephosphorylating cyclin T_1;3_ (Cyct_1;3_) (Qi et al., 2012; Zhang et al., 2012). *GS3*, a major gene controlling GL and grain weight, encodes a putative Gγ protein and inhibits interactions between the Gβ protein RGB1 (G protein γ subunit 1) and two other Gγ proteins to regulate grain size (Sun et al., 2018). *SG3*, which is closely linked to *GS3*, acted as a transcriptional activator and negatively controlled GL in rice *via* a 12-bp insertion in the third exon of NYZ that led to a frameshift and resulted in a premature stop codon (Li Q. P. et al., 2020). *GLW7* encodes the plant-specific transcription factor OsSPL13, which positively regulates GL and yield (Si et al., 2016). *GL7* regulates GL by affecting the expression of two linked genes (Wang et al., 2015). *GL6* encodes a plant AT-rich sequence and zinc-binding (PLATZ) transcription factor; this protein has a premature stop codon and reduces the expression or causes the loss of function of *GL6*, thereby affecting rice GL (Wang et al., 2019).

Although some genes regulating GL have been cloned, few genes have been applied in production, and the overall regulatory networks underlying the associated processes remain poorly understood. With the development of high-throughput sequencing in recent years, high-efficiency, low-cost whole-genome sequencing has become available. Approaches based on bulked segregant analysis (BSA) coupled with whole-genome sequencing (BSA-seq) have been widely applied for mapping several important agronomic-related genes in rice (Zhang et al., 2019), and gene cloning and functional research have become easier. BSA was used to construct two pools by mixing the DNA of individuals with extreme traits in a segregating population (Takagi et al., 2013). By analyzing the differences in single-nucleotide polymorphisms (SNPs) and insertions/deletions (InDels) between the two pools, we can quickly locate the molecular markers tightly linked to a target gene (Klein et al., 2018). This method is appropriate for qualitative traits controlled by one gene and quantitative traits controlled by major QTLs. Currently, BSA-seq, a rapid approach for identifying QTLs, is widely used in research on rice (Yang et al., 2013; Tao et al., 2018; Gao et al., 2019; Liang et al., 2020).

Previously, we obtained the wild rice inbred line Huaye 3 (*Oryza rufipogon* Griff.), which presented a very short GL of only 7.03 mm (Yu et al., 2018). An F_2_ population was then generated from a cross between the cultivar KJ01 with a long GL of 13.33 mm and Huaye 3. A QTL genetic linkage analysis has revealed a novel gene that controls GL and explains 54.85% of the phenotypic variation (Zheng et al., 2020). In this study, BSA-seq was used to narrow the gap of 340 kb on chromosome 3 with an F_2_ population comprising 1,307 individuals. No GL genes were found in this interval, and the novel QTL was named *qGL3.5*. This region included 37 candidate genes according to SNP annotation analysis. Fine mapping was then conducted using 2786 BC_4_F_2_ individuals, and *qGL3.5* was narrowed down to a 24.0-kb genomic interval. Among the genes in this interval, *ORF18* (Gene ID: *LOC_Os03g42790.1*), namely, *qGL3.5*, was found to regulate GL *via* clustered, regularly interspaced, short palindromic repeat (CRISPR)/CRISPR-associated 9 (Cas9). Our study provides a new gene resource for rice grain shape breeding and a starting point for the functional characterization of the wild rice GL gene.

## Materials and Methods

### Plant Materials and Measurements of Characteristics

The wild rice inbred line Huaye 3, which was bred by our laboratory teams (Yu et al., 2018), the cultivar KJ01 provided by Professor Kangjing Liang in Fujian Agriculture and Forestry University, and their F_2_ progeny and BC_4_F_2_ backcross population were used. These materials were grown in the field at South China Agricultural University during the late season in 2015. Ten rows were planted in each of the test plots, and the row spacing was 18 cm × 21 cm. The field management followed conventional procedure in this area. The rice grains were harvested at the mature stage by collecting three major panicles of each plant, after which the materials were put into marking envelopes. Samples of 30 randomly selected filled grains were assessed for GL and grain width, and the length and width of 10 grains placed end to end were measured using a ruler with a precision of ±0.01 mm. The grains of each plant were replicated three times. The average length of a single rice grain was then calculated. The grain weight was determined by weighing 100 filled dry grains using an electronic analytical balance. The weights of three replicates were measured for each plot. The statistical analyses of the data using sample *t*-tests were performed using Prism 8 software.

### Construction of Bulked DNA Pools for Sequencing

Two bulk DNA pools for sequencing were first constructed by selecting individuals exhibiting an extreme phenotype from among 1,307 F_2_ segregating plants. The individuals that produced the 50 longest grains were grouped into the L-pool, and the individuals that produced the 50 shortest grains were grouped into the S-pool among the F_2_ segregating lines.

Total genomic DNA was extracted from young healthy leaves of both parents and 100 selected F_2_ segregating individuals *via* the sodium dodecyl sulfate (SDS) procedure and purified by RNase A. The DNA concentration and quality were estimated with a Nanodrop 2000 UV–Vis spectrophotometer (NanoDrop, Wilmington, DE, United States), and the DNA concentration was diluted to 3 μg/μL; the total DNA was not less than 150 μg. The individual genomic DNA of the 50 L-pool individuals was mixed together equally, constituting the long-seed bulk DNA pool (L-pool), and the individual genomic DNA of the 50 S-pool individuals was mixed together equally, constituting the short-seed bulk DNA pool (S-pool). The genomic DNA of the two bulk DNA pools and both parents was prepared for subsequent high-throughput sequencing.

### Sequencing Data Analysis and Comparison

The four samples were subjected to high-throughput sequencing on an Illumina HiSeq™ 2500 instrument (Illumina, Inc., San Diego, CA, United States) at Biomarker Technologies Corporation in Beijing. Real-time monitoring was performed for each cycle during sequencing, and the percentage of high-quality reads with quality scores greater than Q30 (a quality score of 30, indicating a 0.1% chance of an error and thus 99.9% confidence) among the raw reads and the GC content were calculated for quality control. Afterward, the sequencing data were compared with the sequence of the reference genome of Nipponbare.^1^

### Single-Nucleotide Polymorphism Screening

The detection of SNPs was mainly performed using the GATK software toolkit (McKenna et al., 2010). According to the positioning results of clean reads in the reference genome, Picard^2^ was used for preprocessing, such as marking duplicates, and GATK was used for local realignment and base recalibration to ensure the accuracy of detected SNPs. GATK was then used for SNP detection and filtering, after which a final set of SNPs was obtained.

### Association Analysis

The relative marker abundance in the L-pool was calculated as the number of reads of the maternal allele divided by the number of reads of the paternal allele, whereas in the S-pool, the relative marker abundance was calculated as the number of reads of the paternal allele divided by that of the maternal allele. It was expected that the larger the relative abundance was, the greater the possibility that the marker was associated with GL. SNP index association analysis (Abe et al., 2012) and Euclidean distance (ED) association analysis (Deza and Deza, 2009) were performed in this study, and in the present study, L refers to the KJ01 parent, S refers to the parent of Huaye 3, aa represents the L-pool, and ab represents the S-pool.

#### Single-Nucleotide Polymorphism Index Association Analysis

The idea behind SNP index association analysis was recently published; this analysis is a type of method used to calculate genotype frequency differences between two bulks satisfied by Δ(SNP index). A relatively close marker is associated with traits, while a relatively close Δ(SNP index) is associated with the value 1. The Δ(SNP index) was calculated as follows: Laa is the depth of the aa group derived from L, while Saa indicates the depth of the aa group derived from S. Lab means the depth of the ab group derived from L, while Sab represents the depth of the ab group derived from S.


$$\begin{array}{c} \mathrm{SNP}\mathrm{index}\left(\text{aa}\right)=\text{Laa}/\left(\text{Laa}+\text{Saa}\right)\\ \mathrm{SNP}\mathrm{index}\left(\text{ab}\right)=\text{Lab}/\left(\text{Lab}+\text{Sab}\right)\\ \Delta \text{SNP}\text{index}=\text{SNP}\text{index}\left(\text{aa}\right)-\text{SNP}\text{index}\left(\text{ab}\right) \end{array}$$

#### Euclidean Distance Association Analysis

Euclidean distance association analysis is a method for calculating ED (the quadratic sum root of differences between bulks based on the mutation frequency of the four types of bases). In theory, the higher the ED value is, the closer the object site.

Euclidean distance was calculated as described below. Aaa, Caa, Taa, and Gaa represent the frequency of bases A, C, T, and G, respectively, at a particular site in the large seed bulk. Aab, Cab, Tab, and Gab represent the frequency of bases A, C, T, and G, respectively, at a particular site in the small seed bulk.


$$ED=\sqrt{\begin{array}{c} {\left(A_{aa}-A_{ab}\right)}^{2}+{\left(T_{aa}-T_{ab}\right)}^{2}+{\left(G_{aa}-G_{ab}\right)}^{2}\\ +{\left(C_{aa}-C_{ab}\right)}^{2} \end{array}}$$

In this study, high-throughput sequencing combined with BSA was used to detect SNP tags between the two bulked DNA pools and to rapidly identify marker-intensive hotspots associated with GL in the rice genome.

### Clustered, Regularly Interspaced, Short Palindromic Repeat/CRISPR-Associated 9 Transgene Analysis

Two targets were designed for the potential candidate gene *ORF18 via* the CRISPR-GE website.^3^ The expression cassette vector was constructed by multiple rounds of PCR (Ma et al., 2015). The target sequences were introduced into sgRNA expression cassettes by overlapping PCR. The purified PCR products (sgRNA expression cassettes) were inserted into a pYLCRISPR/Cas9 vector based on the Golden Gate system. The ligated products harboring the sgRNA expression cassettes were directly used to transform *E*scherichia *coli* DH5α competent cells. The CRISPR/Cas9 constructs that were successfully generated were introduced into *Agrobacterium tumefaciens* strain EHA105. *A. tumefaciens*-mediated gene transfer experiments were subsequently carried out in the Huaye 3 background. The results were analyzed *via* the sequential decoding method (see text footnote 3) to identify positive transgenic plants. Three wild-type plants and 23 transformed plants were selected at maturity to measure their GL.

### Histological Analysis

Spikelet hulls from WT and mutant lines were collected after ripening. Glumes with a flat shape were selected and dried completely, and the samples were then laid flat with conductive glue and fixed on the loading table. After solidification, the outer surface of the glumes of the spikelets was sputter-coated with gold and observed with a Verios 460 scanning electron microscope. The cell size was measured using ImageJ software. The cell number and density were calculated along the longitudinal axis.

## Results

### Phenotypic Analysis of Both Parents and Genetic Analysis of Grain Length Within the F_2_ Population

**FIGURE 1:**
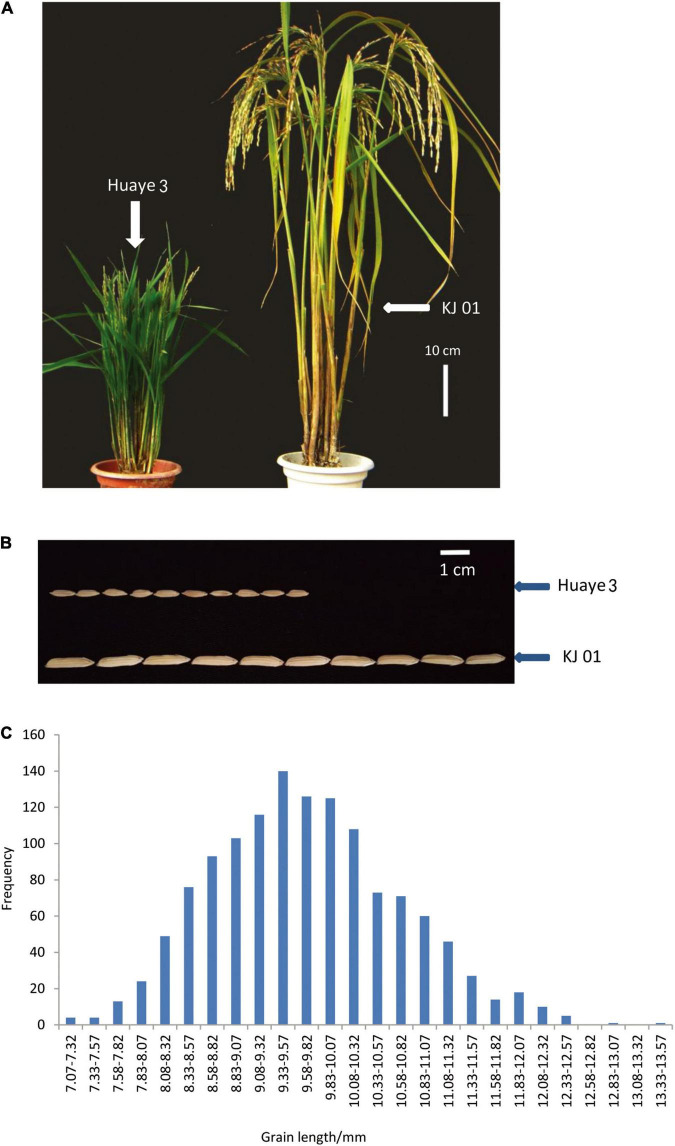
Phenotypic analysis of the parents and frequency distribution of the F_2_ individuals. **(A)** Plant type comparison of KJ01 and Huaye 3. **(B)** GL comparison of KJ01 and Huaye 3. and **(C)** frequency distribution of F_2_ individuals.

All the traits of the wild rice inbred line Huaye 3, including the plant height and GL, remained stable during continuous self-crossing and selective breeding for several years (Figure 1A). The plant height was approximately 63.67 cm, and the GL was approximately 7.03 mm. Huaye 3 was used as one of the parents and crossed with the rice cultivar KJ01, which has a long GL of 13.33 mm. There were significant differences in GL, grain width, plant height, and panicle length between the two parents (Table 1 and Figure 1B). The GL of hybrid F_1_ was 9.40 mm. A genetic analysis of GL for 1,307 separate F_2_ individuals showed that the frequency distribution of GL was continuous, that the means deviated from those of the long-grain parent and that the variation range of GL was very large, from 7.07 to 13.33 mm, in the F_2_ population (Figure 1C). Therefore, the GL of the two parents was a typical quantitative trait controlled by multiple genes.

**TABLE 1:** Phenotypic value of traits for KJ01 and Huaye 3.

	Grain length (mm)	Grain width (mm)	Plant height (cm)	Panicle length (cm)
KJ01	13.33 ± 0.05	3.30 ± 0.23	123.26 ± 1.37	34.75 ± 0.48
Huaye 3	7.03** ± 0.09	1.80** ± 0.15	63.67** ± 0.44	16.63** ± 0.24

“**” indicates a highly significant difference at the P = 0.01 level.

### Qualitative Analysis of the Bulk DNA Sequencing Data

Fifty individuals that produced extremely long grains (11.57–13.33 mm) and 50 individuals that produced extremely short grains (7.07–8.13 mm) from the F_2_ population were selected for preparation of the long-seed pool (L-pool) and short-seed pool (S-pool). In total, 100.60 Gb of raw data were obtained from four samples (KJ01, Huaye 3, the L-pool, and the S-pool), and 100.22 Gbp of clean data were attained after filtering. Of the high-quality data, 55,999,600 reads were obtained from KJ01, and 45,059,434 reads were obtained from Huaye 3. The read numbers for the S-pool and L-pool were 127,409,412 and 106,871,512, respectively. The average percentage between the sample and the reference genome was 96.64%, the average coverage depth was 55×, and the genome coverage was 96.51% (at least one base coverage). Four samples (one each of KJ01, Huaye 3, the L-pool, and the S-pool), with 16.72, 13.46, 31.93, and 38.09 Gb, respectively, were sequenced, and their GC contents were 45.72, 45.54, 46.04, and 46.03%, respectively.

Version RGAP7 of the *Oryza sativa japonica* genome, which comprised 374 Mb and had a GC content of 43.55%, was used as the reference genome. The average depth and Cov-ratio-10x (%) of KJ01 and Huaye 3 were 38x (88.40%) and 31x (90.19%), respectively. Similarly, the average depth and Cov-ratio-10x (%) of the L-pool and the S-pool were 70x (95.23%) and 83x (95.89%), respectively (Supplementary Table 1).

The coverage of the genome can reflect the amount of variation from the reference genome. The more regions that are covered, the more mutation sites can be detected. The coverage was influenced mainly by sequencing depth and distance between the sample and reference genome. The depth of genome coverage affects the accuracy of mutation detection; the higher the coverage depth (non-repetitive sequence region) is, the higher the accuracy of mutation detection is.

Sequencing quality statistics showed that our sequencing data were of high quality, showed good consistency and could be used for subsequent bioinformatics analysis.

### Detection of Single-Nucleotide Polymorphisms Between Samples and Reference Genomes

Single-nucleotide polymorphism mutations can be divided into transition and transversion types. Mutations involving the same type of bases, such as changes between a purine and another purine and between a pyrimidine and another pyrimidine, are called transitions. Mutations involving different types of bases, such as changes between a purine and pyrimidine, are called transversions. Generally, transitions are more likely to occur than transversions, so the transition/transversion (Ti/Tv) ratio is generally greater than one. For diploid species, if a certain SNP site on homologous chromosomes is the same base, the SNP site is referred to as homozygous. If an SNP site on a homologous chromosome involves different types of bases, the SNP site is referred to as a heterozygous SNP.

**FIGURE 2:**
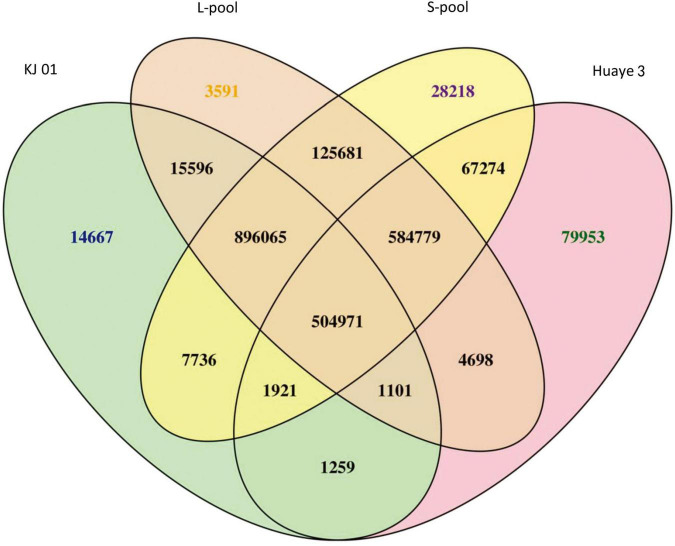
Venn diagram of SNP statistics between samples. The number of varying sites considers only whether the location is the same, not whether the genotype is the same.

In the present study, the higher the number of homozygous SNPs was, the greater the difference between the sample and the reference genome was, and the greater the number of heterozygous SNPs was, the higher the degree of heterozygosity of the sample was. These results are related to the material used. Compared with the number of SNPs in the reference genome, the number of SNPs in KJ01 and Huaye 3 was 1,443,316 and 1,245,956, respectively. For the two bulked pools, the numbers of SNPs in the S-pool and L-pool were 2,216,645 and 2,136,482, respectively (Supplementary Table 2). The number of SNPs common to both the L-pool and S-pool was 2,111,496 (Figure 2). Finally, 2,337,510 SNPs were selected for further analysis after two rounds of sequencing and the exclusion of low-quality fragments.

For SNP index association analysis, 5,201 SNPs with multiple mutations were filtered: 772,175 SNPs were the same in the two bulk pools, 23,119 SNPs had a read number of less than four, and 311,069 SNPs were non-existent in the parents. These SNPs were all filtered out. A total of 1,225,946 SNPs were ultimately selected for further analysis.

For ED association analysis, 45,000 SNPs with read support less than four in any bulk pool were filtered out, 513,705 identical SNPs between bulk pools were filtered out, and 4,806 SNPs with multiple alignments were filtered out. Finally, 1,773,999 SNPs were selected for ED association analysis.

### Single-Nucleotide Polymorphism Index and Euclidean Distance Association Analysis

**FIGURE 3:**
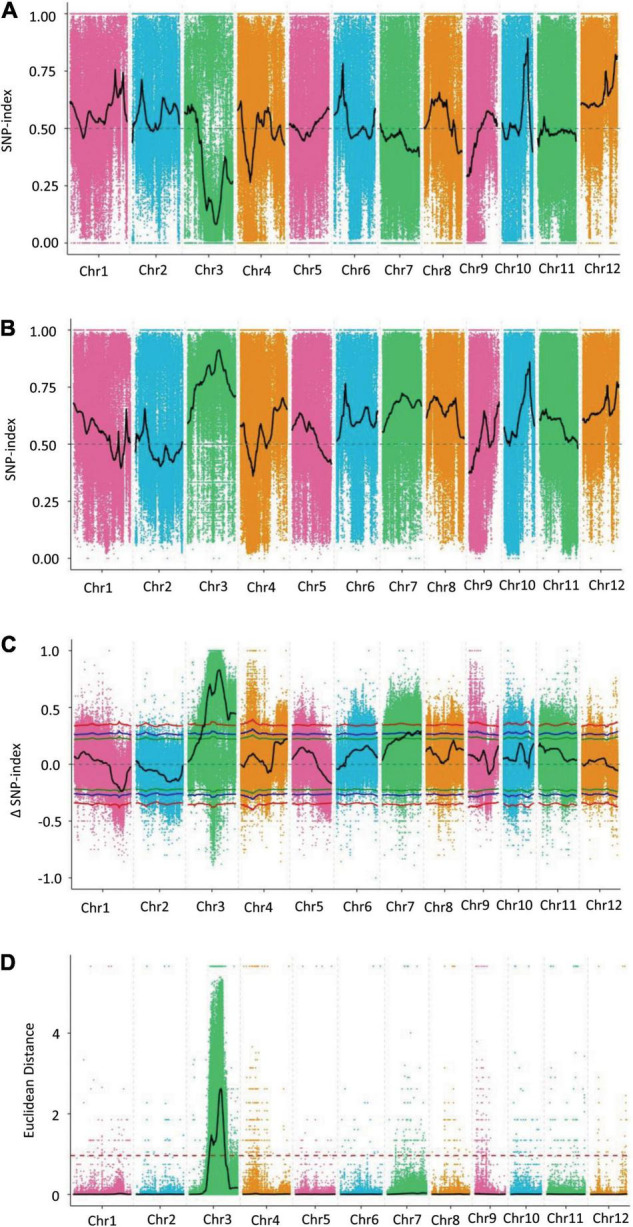
Distribution diagram of the associated values for the SNP index and ED on chromosomes. **(A)** Distribution diagram of the associated value for the SNP index of the L-pool. **(B)** Distribution diagram of the associated value for the SNP index of the S-pool. **(C)** Distribution diagram of the Δ(SNP index) for the S-pool and L-pool. **(D)** Distribution diagram of the associated value for ED. The colored dots represent the calculated SNP index or the ED value of each SNP site, and the black lines show the fitted SNP index, Δ(SNP index) value, or ED value. In C, the red line shows the association threshold value of 99%, the blue line shows the association threshold value of 95%, and the green line shows the association threshold value of 90%. In **(D)**, the red dashed line represents the significance association threshold of 0.9650. A higher ED value indicates a better association effect.

Two BSA-seq analysis methods, the SNP index and ED, were used to identify the candidate genomic regions associated with GL. After filtering, a total of 1,225,946 SNPs were used for association analysis through the SNP index method. An SNP index of the L-pool and S-pool was calculated for each identified SNP in the genome, and an average SNP index was computed for each 1 Mb interval *via* a 10-kb sliding window (Figures 3A,B). By combining the SNP index information of the L-pool and S-pool, we calculated the Δ(SNP index), and trends in the Δ(SNP index) were visualized by means of a sliding window. Using the association threshold of 99%, we identified only one genomic region distributed on chromosome 3 that was significantly correlated with GL (Figure 3C). The length of the region was 25.53 Mb in the interval from 10,880,000 to 36,410,000 bp and contained 4,734 genes.

A total of 1,773,999 SNPs were used for association analysis through the ED method. The quadratic power of ED was used as the correlation value to eliminate background noise, and the DISTANCE method was used to fit the ED value. The association threshold was calculated as 0.9650 by adding 3 standard deviations to the median for all the fit values. The distribution of the correlation value for all chromosomes was examined, but only chromosome 3 showed a region that crossed the significance association threshold; *qGL3.5* was mapped in the interval from 15,080,000 to 27,070,000 bp on chromosome 3 (Figure 3D). Because the genomic region of interest was too large to carry out subsequent verification, the 99 quantile method was conducted to further analyze the ED results, and four associated regions on chromosome 3 closely correlated with the GL trait were obtained. By analyzing the four associated regions, we found that the first region was 0.03 Mb in the interval from 23,160,000 to 23,190,000 bp and contained 6 candidate genes. The second region had only one gene at the 23,210,000 bp position, and the third region was 0.11 Mb in the interval from 23,250,000 to 23,336,000 bp and contained 16 genes, and the fourth region was 0.20 Mb in the interval from 23,830,000 to 24,030,000 bp and contained 34 genes. The total length of these regions was 0.34 Mb, and they contained a total of 57 genes closely correlated with the GL trait (Supplementary Table 3).

By combining the association results of the SNP index and ED methods, we found that the intersections of the genomic regions were consistent with the associated regions identified *via* the ED method. The 0.34 Mb region of chromosome 3, which contained 57 genes, identified *via* BSA-seq was the “hotspot” for GL in rice. There were no genes controlling GL reported in these genomic regions. The differences between the parents regarding the SNPs within exon regions in these 57 genes were analyzed. There were a total of 246 non-synonymous SNPs involving 37 genes.

These results in turn allowed us to narrow the genomic region of the QTL linkage analysis, which identified the major QTLs as being within the interval of markers PSM379-RID24455, spanning 7.4 Mb (Zheng et al., 2020).

### Fine Mapping of *qGL3.5*

**FIGURE 4:**
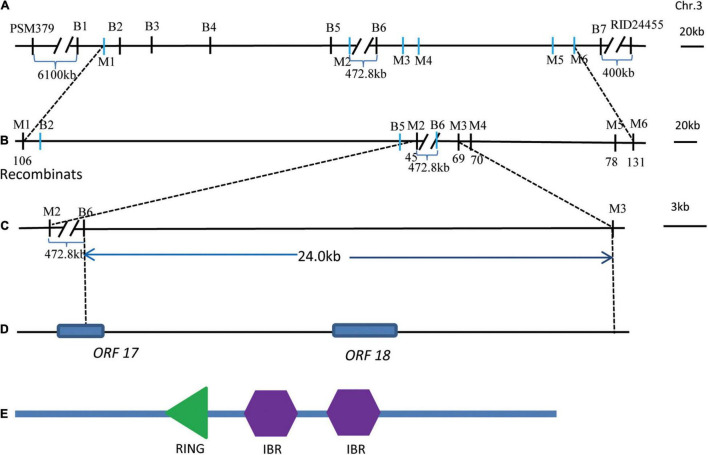
Fine mapping of *qGL3.5*. **(A)** Regions associated with GL identified *via* the ED method of BSA. B1 to B2 were the first region, B3 was the second association region, B4 to B5 was the third association region, and B6 to B7 was the fourth association region. **(B)** Genotype and phenotype analysis of the associated regions determined using newly developed Indel markers. M1–M6 were Indel markers. **(C)** *qGL3.5* was mapped to the genomic interval between the flanking markers M2–M3. Because the region from B5 to B6 was not the associated regions, *qGL3.5* was mapped to the genomic interval between B6 and M3, which was narrowed down to a 24.0-kb region. **(D)** There was only one complete annotated gene, *ORF18*, and part of the coding region of another gene, *ORF17*, with non-synonymous SNPs between KJ01 and Huaye 3 based on BSA-seq analysis. **(E)** Domains for the candidate *ORF18*.

To further fine map *qGL3.5*, KJ01 was used as the recurrent parent, and a BC_4_F_2_ backcross population was generated to narrow down the locus to a small region. Six pairs of polymorphic Indel markers (M1–M6) were developed in the preliminarily mapped interval by BSA based on the sequencing data of KJ01 and the wild rice mutant (Figure 4B and Supplementary Table 4). A total of 2786 BC_4_F_2_ individuals were used for fine mapping of *qGL3.5*. Their genotypes were analyzed using six pairs of newly developed PCR markers. Linkage analysis of the phenotype was performed with marker genotypes. Finally, *qGL3.5* was mapped to the genomic interval between flanking markers M2 and M3. BSA-seq analysis showed that the genomic interval from B5 to B6 did not contain the associated regions (Figure 4A). Finally, *qGL3.5* was mapped to the genomic interval between B6 and M3, which was narrowed down to a 24.0-kb region (Figure 4C). Within all 37 genes with non-synonymous SNPs between KJ01 and Huaye 3 in BSA-seq analysis, there was only one complete annotated gene, *ORF18*, and part of the coding region of another gene, *ORF17* (Figure 4D). Thus, *ORF18* (Gene ID: *LOC_Os03g42790.1*) was selected as the candidate gene, named *qGL3.5*, and its function was verified. *ORF18* comprised five exons and four introns and encoded an IBR-RING zinc-finger-related protein with one really interesting new gene (RING) and two in between ring finger (IBR) domains (Figure 4E). Genomic sequencing of *ORF18* showed that there were four non-synonymous SNPs in the coding region between KJ01 and Huaye 3, which resulted in four amino acid changes. Compared with Huaye 3, six positions within the promoter region of *ORF18* were changed in KJ01, including 1-bp deletion at four positions and 2-bp deletion at two position (Supplementary Table 5).

### Functional Analysis of *ORF18* Using Clustered, Regularly Interspaced, Short Palindromic Repeat/CRISPR-Associated 9 Gene-Editing Technology

**FIGURE 5:**
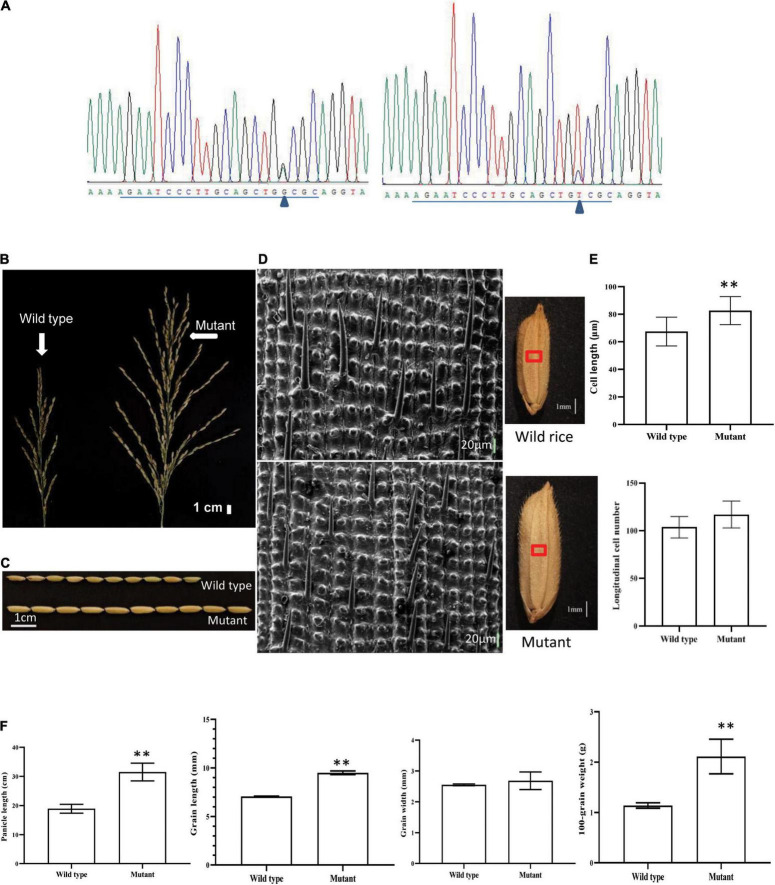
Phenotypic and genotypic analyses of T_0_ and T_1_ generations after the knockout of *ORF18* of Huaye 3 using CRISPR/Cas9 editing technology. **(A)** Target site 1 derived from mutants generated two edited types: one was a base insertion of G or A, and the other was a base insertion of T or C; “Δ” indicated the variant base, and the underlined sequences are the target site. **(B)** Spike shape analysis of the wild type and mutant. **(C)** Comparison of the grain length between the wild type and mutant. **(D)** Comparison of the longitudinal cells of the grain outer glumes between the wild type and mutant. **(E)** Comparison of the cell length and longitudinal cell numbers between the wild type and mutant. **(F)** Analyses of the differences in the panicle, grain length, grain width, and 100-grain weight between the wild type and mutant. “^**^” indicates a highly significant difference at the *P* = 0.01 level.

The molecular function of *ORF18* was verified *via* CRISPR/Cas9 gene-editing technology. The targets of 7 of the 16 plants were edited after *ORF18* was knocked out in Huaye 3. *ORF18* exhibited three gene-editing types in the seven plants (Table 2). Among them, type II and type III were the main editing types. In the first target site, all the mutants involved a base insertion of A and G or T and C (Figure 5A). In the second target site, most of the mutants consisted of the deletion of ten base pairs or a base insertion of T. Remarkably, the GL and spike of the seven edited T_0_ plants became longer, and the panicle produced more primary and secondary branches (Figures 5B,C). The T_0_ plants of the three edited types were then grown in T_1_ lines. The changed traits remained stably inherited in the T_1_ plants. The GL increased by approximately 2.2 mm, and the panicle increased by approximately 12.18 cm compared with that of the wild type. The difference in the GL and panicle between the knocked mutant and wild type was very significant, which resulted in an 85.09% increase in the 100-grain weight of the mutant, but the difference in the grain width between the mutant and the wild type was not obvious (Figure 5F).

**TABLE 2:** Gene-editing types for two target sites of *ORF18* in T_0_ plants.

No. of plants	Target site 1	Target site 2	Type
#1	+A or G	−2 bp	I
#2	+A or G	−10 bp	II
#3	+A or G	−10 bp	II
#4	+A or G	−10 bp	II
#5	+T or C	+T	III
#6	+T or C	+T	III
#7	+T or C	+T	III

*“+” indicates insertion; “−” indicates deletion.*

### *qGL3.5* Regulated the Longitudinal Cells of the Grain Outer Glumes

The division and elongation of the spikelet hull cells determine the grain size; thus, the outer spikelet hull surfaces of the mutant and the wild type were observed by scanning electron microscopy. In the same field of view, the distance between the two adjacent protrusions was longer in the mutant sample than in the wild type (Figure 5D). The length of longitudinal cells was 29.71% higher in the mutant, and this difference was very significant; however, the difference in the numbers of longitudinal cells was not obvious (Figure 5E). These results showed that *qGL3.5* regulated GL by increasing the length of longitudinal cells.

## Discussion

### Wild Rice Contains Many Valuable Genes

As research has advanced, the genetic resources derived from cultivated rice have become increasingly narrower. Because it has excellent genes associated with resistance and yield, wild rice has become a good candidate to explore valuable genes to enhance rice productivity (Rahman et al., 2007; Guo et al., 2009). To date, an increasing number of finely mapped or cloned genes from wild rice have been successively reported.

*GAD1*, derived from *O. rufipogon*, encodes a small secretory signal peptide and regulates both GL and awn development (Jin et al., 2016). *qHD7.2*, which delays rice flowering and shortens panicle length under long-day conditions, was identified in *O. rufipogon* Griff. (Li J. et al., 2018). Wang et al. (2019) cloned *GL6*, which controls rice GL and is derived from *O. rufipogon* W1943; *GL6* encodes a PLATZ transcription factor. *qGCA1* derived from *Oryza longistaminata* significantly improves the general combining ability of plant height, spikelet number, and yield per plant and was delimited to an interval of approximately 72 kb on chromosome 1 (Fan et al., 2019). *ORF12* from *Oryza minuta* was identified as a candidate gene (*TGW12*) that controls the 1,000-grain weight of rice (Li X. Q. et al., 2020). In our study, by using the BSA method and CRISPR/Cas9 gene-editing technology, we identified a new gene, *qGL3.5*, that controls rice GL and is derived from *O. rufipogon*.

### *qGL3.5* Is a Novel Regulator of Grain Length in Rice

The molecular mechanisms behind GL regulation are diverse. These GL regulators are reportedly involved in multiple signaling pathways, which mainly include G protein signaling, the mitogen-activated protein kinase (MAPK) signaling pathway, the ubiquitin–proteasome pathway, phytohormone signaling, and transcriptional regulatory factors (Li N. et al., 2018). *GS3* was the first molecularly characterized QTL for grain size, which regulated GL by combining the G protein β subunit signaling of DEP1 and GGC2 (Sun et al., 2018). The overexpression of *qGL3 via* OsGSK3 phosphorylation inhibits BR signaling and produces a BR loss-of-function phenotype (Gao et al., 2019). *qGL3* also promotes OsGF14b protein phosphorylation, induces the interaction of OsGF14b and OsBZR1, and negatively regulates BR signaling (Gao et al., 2021). OsARF6 regulates the expression of OsAUx3 by binding directly to its promoter, whereas OsARF6 is posttranscriptionally regulated by miR167a, resulting in increasing GL and GW (Qiao et al., 2021).

Previous studies have shown that the expression levels of extensors are negatively correlated with the degree of cell extension, and increasing the expression levels of extensors may promote an increase in the cell density in local tissues or organs in which these genes are expressed (Roberts and Shirsat, 2006), which would result in an increase in GL. For example, *GS6* encodes a GRAS domain protein and affects lemma development by regulating the number of cells in glumes and thus negatively regulating the size of rice grains (Sun et al., 2013). On the other hand, some genes controlling GL regulated it by lengthening the longitudinal cells of the rice hull. *GL7* positively regulates longitudinal cell elongation *via* tandem duplication of a 17.1-kb segment and thus increases GL (Wang et al., 2015). In our study, we identified a gene, *ORF18*, that negatively controlled GL by promoting longitudinal cell elongation in outer glumes.

*qGL3.5* encodes an IBR-RING zinc-finger protein with one RING and two IBR domains. Proteins containing zinc finger domains and RING-finger domains were found to play important roles in eukaryotic cells by regulating different signal transduction pathways and biological processes, such as plant growth development and resistance (Yilmaza and Mittlera, 2008; Sun et al., 2019; Zhao et al., 2021). Duan et al. (2020) reported a Cys2/His 2 zinc finger protein, ZFP207, that acts as a transcriptional repressor of *OsGA20ox2* and negatively regulates plant height and GL in rice. *GW2*, which encodes a RING-type E3 ubiquitin ligase, regulates grain size by restraining cell division and reducing the width of the glume (Bai et al., 2010; Choi et al., 2018). *CLG1* (Chang L Geng1-1), which encodes an E3 ligase containing a CHY zinc-finger domain and a C3H2C3-type RING domain, regulates GL by directly interacting with GS3 (Kobayashi et al., 2013; Yang et al., 2021). However, the regulation of GL by IBR-RING zinc-finger protein has not been previously investigated.

### *qGL3.5* Significantly Enhances Rice Yield

Increasing the rice yield is urgently needed to support the rapid growth of the global population (Li N. et al., 2018). Grain size is an important agronomic trait for improving the yield of rice. *OsSGL* was overexpressed in 93-11, and the transgenic lines showed larger panicles than the wild-type 93-11. The grains of 93-11-OE were on average 24.8% longer, 8.6% narrower and 16.3% heavier, which theoretically lead to an average 46.2% increase in the grain yield per panicle (Wang et al., 2016). *OsNAC23* was overexpressed in Nanjing 46, and the transgenic lines exhibited increased panicle numbers, panicle size, and grain size and showed 11.46–12.56% yield increases compared with Nanjing 46 (Li et al., 2022). In our study, *qGL3.5* was knocked out in Huaye 3, and the grain size and panicle shape of the edited transgenic plants showed obvious changes. The GL and panicle of the transgenic plants were increased by approximately 2.2 mm and approximately 12.18 cm compared with those of the wild type, respectively. These increases directly resulted an 85.09% increase in the 100-grain weight of the mutant and an enhancement in the rice yield. Therefore, the loss-of-function mutant *qGL3.5* could be applied in production to increase the rice yield.

## Conclusion

In this study, BSA-seq was used to rapidly and successfully explore candidate genes that control GL in rice. By combining the association results of the ED and SNP index methods, we found that four associated regions on chromosome 3 were closely correlated with the GL trait. In these genomic regions, no genes controlling GL have been reported, and the novel QTL was named *qGL3.5*. The total length of these regions was 0.34 Mb and contained 57 genes. A total of 246 non-synonymous SNPs between the parents were found in the exon regions of 37 genes. By fine mapping using 2786 BC_4_F_2_ individuals, *qGL3.5* was narrowed down to a 24.0-kb genomic interval outside the interval identified in BSA-seq analysis. This region contained one complete annotated gene, *ORF18*, and part of the coding region of *ORF17*. Using CRISPR/Cas9 gene-editing technology, *ORF18* was verified to negatively regulate GL and was the target gene of *qGL3.5*. *ORF18* regulated GL by longitudinal cells of the grain outer glumes.

## Data Availability Statement

The datasets presented in this study can be found in online repositories. The names of the repository/repositories and accession number(s) can be found below: https://www.ncbi.nlm.nih.gov/, PRJNA729163.

## Author Contributions

LW: conceptualization, methodology, data analysis, and writing—original draft. XL: conceptualization, methodology, writing—review and editing, and developing Huaye 3. YL, HZ, YZ, FB, and SD: performing the experiments. ZC and JW: writing—review and editing. All authors contributed to the article and approved the submitted version.

## Conflict of Interest

The authors declare that the research was conducted in the absence of any commercial or financial relationships that could be construed as a potential conflict of interest.

## Publisher’s Note

All claims expressed in this article are solely those of the authors and do not necessarily represent those of their affiliated organizations, or those of the publisher, the editors and the reviewers. Any product that may be evaluated in this article, or claim that may be made by its manufacturer, is not guaranteed or endorsed by the publisher.

## Footnotes

1
https://www.ncbi.nlm.nih.gov/genome/?term=txid4530[orgn]

2
http://sourceforge.net/projects/picard/

3
http://skl.scau.edu.cn/
