# Supplementary material for: Identification of qGL3.5, a Novel Locus Controlling Grain Length in Rice Through Bulked Segregant Analysis and Fine Mapping
Source: Front Plant Sci. 2022 Jun 15;13:921029. doi: 10.3389/fpls.2022.921029 (PMC9240483; doi:10.3389/fpls.2022.921029)
Supplement: Supplementary file 1 [file Data_Sheet_1.PDF]

Table S1 Statistical analysis of the sequencing data for each sample

Sample	Clean Base	Q30(%)	GC(%)	Ave depth	Cov-_ratio_10X(%)	Mapped(%)	Properly mapped(%)
KJ01	1672855240	94	45.72	38	88.4	97.52	90.06
Huaye 4	13466564590	93.8	45.54	31	90.19	97.77	91.14
L-pool	31934634710	93.8	46.04	70	95.23	95.51	88.31
S-pool	38091366320	93.9	46.03	83	95.89	95.75	88.57

Table S2 SNPs analysis of the four samples

Sample	SNP numbers	Transition	Transversion	Ti/Tv	Heterozygosity	Homozygosity	Het-ratio (%)
KJ01	1443316	1036169	407147	2.54	175341	1267975	12.14
Huaye 4	1245956	890859	355097	2.50	251407	994549	20.17
L-pool	2136482	1531309	605173	2.53	1545825	590657	72.35
S-pool	2216645	1590470	626175	2.53	1765226	451419	79.63

Table S3. Association regions for GL according to ED analysis.

Chromosome ID	Start (bp)	End (bp)	Size (Mb)	Gene number
Chr3	23,160,000	23,190,000	0.03	6
Chr3	23,210,000	23,210,000	0.00	1
Chr3	23,250,000	23,360,000	0.11	16
Chr3	23,830,000	24,030,000	0.20	34

Table S4 Primer sequences of the newly developed Indel markers for fine mapping

Indel markers	Primer sequences (from 5' terminal to 3' terminal)
M1	F: TTCTGGAAGTACACATACCCAAA<br>R: GCCCAATACGTCGTTGGTTT
M2	F: GCGCAAGTAAATCATAGTCCACA<br>R: GCATGCATGTAACATTTATCGCTG
M3	F: AGCCCACCCTAGCCTATGTC<br>R: AATGAAGGGGCAAGGAAGGT
M4	F: TGCCAGGCCTAATTAAGAGCA<br>R: CGGCCAAAGTGGTAGTAATGG
M5	F: AGCTACGCCTAGATTGCCAC<br>R: GACGCGGAGGGGATTAAGG
M6	F: AGAGTAGTGCTAAGAATGTGTTTGT<br>R: AGCTGCCTCTGATATGTCGC

Table S5 Genomic sequence differences in the coding region and promoter region of *qGL3.5* between KJ01 and Huaye 3.

Genomic sequence	Position	KJ 01	Huaye 4	Codon	Amino acid
Coding region	+2124	T	G	CTG→CGG	Leu→Arg
	+2187	G	A	TGC→TAC	Cys→Try
	+2196	C	T	GCC→GTC	Ala→Val
	+2886	C	A	CAC→AAC	His→Asn
Promoter region	-629	AA	--		
	-999	TT	--		
	-1068	G	--		
	-1188	T	--		
	-1259	T	--		
	-1281	T	--		

Note: "--" represents bases deletion
